# Clinical, Neuroimaging, and Neurophysiological Findings in Children with Microcephaly Related to Congenital Zika Virus Infection

**DOI:** 10.3390/ijerph16030309

**Published:** 2019-01-23

**Authors:** Maria-Lucia C. Lage, Alessandra L. de Carvalho, Paloma A. Ventura, Tania B. Taguchi, Adriana S. Fernandes, Suely F. Pinho, Onildo T. Santos-Junior, Clara L. Ramos, Cristiana M. Nascimento-Carvalho

**Affiliations:** 1 Post-graduate Programme in Health Sciences, Federal University of Bahia School of Medicine, Praça XV de Novembro—Largo do Terreiro de Jesus, 40025-010 Bahia, Brazil; nascimentocarvalho@hotmail.com; 2 Pediatric Rehabilitation Centre, Salvador Hospital, SARAH Network of Rehabilitation Hospitals, Avenida Tancredo Neves, Caminho das Árvores, Salvador, 41820-900 Bahia, Brazil; 13110@sarah.br (A.L.d.C.); 701064@sarah.br (P.A.V.); 11786@sarah.br (T.B.T.); 400868@sarah.br (A.S.F.); 701053@sarah.br (S.F.P.); 3 Diagnostic Imaging Department, Salvador Hospital, SARAH Network of Rehabilitation Hospital, Avenida Tancredo Neves, Caminho das ÁrvoresSalvador, 41820-900 Bahia, Brazil; 700991@sarah.br; 4 Bahiana School of Medicine, Bahiana Foundation for Science Development, Dom João VI, Salvador, 40290-000 Bahia, Brazil; ccclr1@gmail.com (C.L.R.); 5 Departament of Paediatrics, Federal University of Bahia School of Medicine, Praça XV de Novembro—Largo do Terreiro de Jesus, 40025-010 Bahia, Brazil

**Keywords:** Zika virus, microcephaly, neuroimaging, neurologic examination

## Abstract

Zika virus (ZIKV) infection appeared in Brazil in 2015, causing an epidemic outbreak with increased rates of microcephaly and other serious birth disorders. We reviewed 102 cases of children who were diagnosed with microcephaly at birth and who had gestational exposure to ZIKV during the outbreak. We describe the clinical, neuroimaging, and neurophysiological findings. Most mothers (81%) reported symptoms of ZIKV infection, especially cutaneous rash, during the first trimester of pregnancy. The microcephaly was severe in 54.9% of the cases. All infants presented with brain malformations. The most frequent neuroimaging findings were cerebral atrophy (92.1%), ventriculomegaly (92.1%), malformation of cortical development (85.1%), and cortical–subcortical calcifications (80.2%). Abnormalities in neurological exams were found in 97.0% of the cases, epileptogenic activity in 56.3%, and arthrogryposis in 10.8% of the infants. The sensorineural screening suggested hearing loss in 17.3% and visual impairment in 14.1% of the infants. This group of infants who presented with microcephaly and whose mothers were exposed to ZIKV early during pregnancy showed clinical and radiological criteria for congenital ZIKV infection. A high frequency of brain abnormalities and signs of early neurological disorders were found, and epileptogenic activity and signs of sensorineural alterations were common. This suggests that microcephaly can be associated with a worst spectrum of neurological manifestations.

## 1. Introduction

Zika virus (ZIKV) was identified in Brazil in 2015 [1], after having appeared in Africa seven decades ago [2]. The first outbreak occurred in Micronesia in 2007 [3]. Infection in humans has always been associated with a common exanthematous disease with limited complications [4]. However, reports from the Brazilian epidemic indicated that ZIKV infection during pregnancy was associated with microcephaly and severe birth disorders [5,6].

Evidence of the transplacental transmission of ZIKV was provided through the detection of viral proteins and viral RNA in placental tissue samples from expectant mothers infected at different stages of pregnancy [7]. This caused great concern about the impact on public health. The scientific community was challenged to better understand the possible changes in the epidemic potential of the ZIKV, its neurotrophic mechanism, and the different spectra of its clinical manifestations in humans. Congenital Zika Syndrome (CZS) was recognised by severe microcephaly, decreased brain tissue with subcortical calcifications, damage to the back of the eye, clubfoot or arthrogryposis, and hypertonia [8]. ZIKV was included in the list of infectious agents that cause congenital malformations, with a spectrum of neurological manifestations beyond microcephaly. Neurological manifestations were described as multiple and severe brain abnormalities [5,9,10], arthrogryposes [11,12,13], hearing loss [14], seizures [12,13,15], pyramidal/extrapyramidal syndrome [12,13], retinal and/or optic nerve alterations [16], and dysphagia [17].

In this study, we aimed to describe the clinical, neurophysiological, and neuroradiological characteristics of children with congenital microcephaly diagnosed at birth and potentially associated with congenital ZIKV infection.

## 2. Methods 

### 2.1. Paticipants and Procedures

This was a retrospective cross-sectional study conducted at Rehabilitation Centre in Salvador, Northeastern Brazil, from November 2015 to September 2016. This centre provides evaluation and follow-up for children referred with neurodevelopmental disorders. All infants were evaluated by an interdisciplinary team according to standardised procedures.

The criteria of inclusion were children born with microcephaly, whose mothers resided in an area affected by ZIKV epidemic during pregnancy and had clinical symptoms of ZIKV during any trimester of pregnancy, mainly cutaneous rash, and negative results for other congenital infections (HIV, cytomegalovirus, toxoplasmosis, rubella, syphilis, and hepatitis B and C). Microcephaly was defined as a head circumference (HC) of 2 standard deviations (SD) below the mean for age, sex, and gestational age at birth [18]. Severe microcephaly was diagnosed when HC was <−3 standard deviations below the mean for sex, age and ethnicity [19]. Children who presented severe complications related to prematurity whose image alterations did not present a pattern suggestive of congenital infection, suffered perinatal asphyxia, or had other signs suggestive of a genetic syndrome, and those whose data were incomplete in the medical chart were excluded. Children whose mothers presented with a history of gestational risk factors for congenital malformation, such as syphilis, toxoplasmosis, rubella, cytomegalovirus (STORC), and dengue diagnosis during pregnancy, as well as illicit drug use, were also excluded. 

Possible congenital ZIKV Infection was considered for infants with clinical findings consistent with CZS, regardless of maternal testing results [20].

### 2.2. Assessment

Admission occurred after online registration, where specific registration for infants with suspected microcephaly was available. Paediatricians performed the first evaluation. After microcephaly was diagnosed based on the definition mentioned above, the infants were referred to be evaluated by an interdisciplinary-trained team (paediatricians, physiotherapists, nurses, psychologists, and speech therapist). All evaluations were standardised. Data on prenatal and perinatal history, major complaints, and childcare routine (behaviour eating difficult, irritability/impatient cry, and sleep disorders) were collected from the medical chart. Data from physical and neurological examination included muscle tone, reflexes, involuntary movements, and motor and cognitive development markers. The evaluation of neurodevelopmental milestones was performed considering the milestone parameters expected for the infant’s age [21]. All data were registered in predefined forms.

The detection of specific antibodies against distinct congenital infections (Syphilis, Toxoplasmosis, Cytomegalovirus, Rubella, and Dengue) was performed in all infants. Enzyme-Linked Fluorescent Assay (ELFA) was the method employed to rule out cytomegalovirus infection, rubella, and toxoplasmosis. Immunochromatographic rapid testing was used to rule out dengue infection, and the Venereal Disease Research Laboratory test (VDRL) was used to screen syphilis in all serum samples.

All infants underwent neuroimaging studies. The brain computed tomography (CT) was made using a multislice CT scanner (CT 64 channel, Phillips Brilliance, Cleveland, OH, USA) without contrast. The magnetic resonance imaging (MRI) was performed (Signa HDxt 1.5T MRI Scanner, GE Healthcare, Wauskesha, WI, USA) without contrast. The MRI sequences available for analysis were T1-weighted imaging, T2-weighted imaging, T2 * gradient echo, and diffusion-weighted imaging. An expert neuroradiologist read and described all neuroimaging exams using predefined forms.

Videoeletroencephalograms (VEEGs) were performed in all infants (Neuroworkbench Version 07-01, Nihon Koden Coporation, Tokyo, Japan). VEEG electrodes were positioned with the electroencephalographic cap according to the international 10–20 system. All the VEEG recordings were performed during spontaneous sleep and were evaluated by a qualified neurophysiologist. Auditory evaluation was carried out by a screening test which consisted of measurement of the short-latency auditory brainstem response (ABR) to click stimuli. The ABR was considered to be normal when wave V was identified in two consecutive averaged waveforms at 35 decibels normal hearing level (dB nHL). The visual screening was analysed by visual evoked potential (VEP) (Viking EDX Version 20.1, Natus Neurology Incorporated, Middleton, WI, USA). Several stimuli were obtained with pulses of 2.0–3.0 Hz. The averages were achieved by summing up 100 responses. VEP waveforms were evaluated using a baseline that appeared 100 ms after pulse stimulus onset and were analysed by experienced neurophysiologists in real time.

### 2.3. Ethics Principles

Ethical approval was obtained from the Ethics Committee of the SARAH Network Rehabilitation Hospitals (Protocol Number: 1.633.570). 

### 2.4. Statistical Analysis 

The data were collected from the electronic medical record and entered into a database using Microsoft^®^ Access Program (Microsolft Acess 2010 Version 14.0.6029.1000, Microsoft Corporation, Redmond, WA, USA). A descriptive analysis of variables was performed using statistical package SPSS 21.0™ (Version 21.0, IBM SPSS Statistics, Armonk, NY, USA).

## 3. Results

In the study period, 181 infants were identified, out of which 36 (19.9%) had other causes of congenital microcephaly and 43 (23.8%) had incomplete medical charts. Therefore, this study group comprised 102 infants with microcephaly diagnosed at birth and gestational ZIKV clinical symptoms. Microcephaly was diagnosed by ultrasound during prenatal care in 67 (65.7%) cases. Severe microcephaly was identified in 56 (54.9%) infants, the mean HC at birth was 28.6 ± 1.7 cm, and the mean HC upon recruitment was 33.1 ± 4.4 cm. However, the infants were born without other serious neonatal complications (Table 1 and Table 2).

The mean age when mothers gave birth was 28.3 ± 5.9 years and one (1.0%) mother had a dizygotic twin pregnancy, out of which one was identified with microcephaly. We observed that 59 (57.9%) lived in the Salvador metropolitan area, 40 (39.2%) lived in the countryside, and 3 (2.9%) came from other Brazilian Northeastern states. All mothers reported ZIKV clinical symptoms (100% rash, 49.0% arthralgia, 41.2% fever, 15.7% headache) during early pregnancy, with 83 (81.4%) during the first trimester (1–13 weeks) and 19 (18.6%) during the second trimester (14–26 weeks). The mothers received regular prenatal care and 39.2% reported problems during pregnancy, such as urinary tract infection (50.0%), vaginal bleeding (35.0%), arterial hypertension (32.5%), and gestational diabetes mellitus (5.0%). 

No infant had positive IgM antibodies against cytomegalovirus, dengue, rubella, toxoplasmosis, or positive VDRL. 

**Figure 1:**
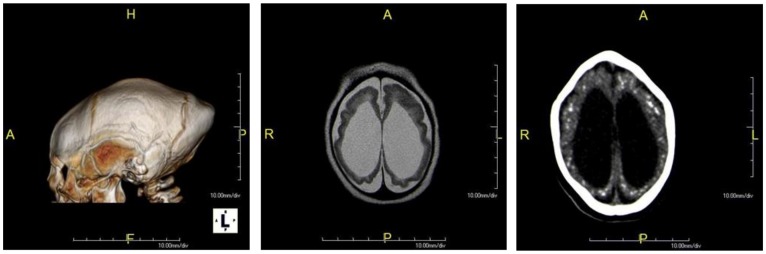
Neuroimage findings in infants with Congenital Zika Syndrome (CZS). 3D CT image of a 3-month-old infant with probable CZS evidencing microcephaly and prominent occiput (**A**). Axial T2/FSE (Fast Spin Echo) MR image of a 4-month-old infant with possible CZS demonstrating ventriculomegaly, diffuse cerebral atrophy, and malformations of cortical development with a simplified gyral pattern (**B**). Axial CT image without contrast of a 4-month-old infant with possible CZS evidencing microcephaly, ventriculomegaly, and bilateral cortical and subcortical calcifications (**C**).

All infants had brain abnormalities detected in the neuroimaging. The VEEG was abnormal in 64.8% of the cases, ABR in 17.3%, and VEP in 14.1% (Table 3). Frequent disorders were found in the neurological evaluation of these infants: hypertonia/spasticity (90.1%), hyperreflexia (73.3%), and neurodevelopmental milestones delay (92.8%) (Table 3). Arthrogryposis was present in 11 (10.8%) of the cases. The main neuroimage abnormalities are shown in Figure 1. Some of the caregivers of these children (39.0%) reported in the first evaluation with the team different problems regarding the child’s behaviour, such as eating challenges (36.3%), irritability/impatient cry (27.5%), and sleep challenges (9.8%).

## 4. Discussion

The results of this study showed that all infants with gestational exposure to ZIKV presented microcephaly with craniofacial disproportion and a high frequency of brain abnormalities. Most of them had serious neurological findings (97.0%), frequent epileptogenic activity (56.3%), and signs suggestive of sensorineural alterations: hearing loss in 17.3% of the cases and visual impairment in 14.1%. Additionally, the caregivers reported different behavioural problems, like eating difficulties (36.3%), irritability/impatient cry (27.5%), and sleep disorders (9.8%). Recently, studies showed that children with CZS presented low performance in cognitive and motor development [22,23]. 

ZIKV during pregnancy shares some features with other congenital infections, but with a spectrum that is substantially different. Microcephaly with craniofacial disproportion was the first sign recognised, followed by brain malformations [5,6]. The estimated risk of microcephaly in children born from women who had symptoms of ZIKV infection during pregnancy ranges from 6.0% [10] to 42% [5].

The neuroimaging findings in our population corroborate the severity of injuries that this infection can cause in the developing brain. The most frequent abnormalities were atrophy (92.1%), ventriculomegaly (92.1%), malformations of cortical development (85.1%), and cortical and subcortical calcifications (80.2%). These findings are in line with those of other publications [9,10,12,13,24]. In this context, a study showed that the findings of neuroimaging between groups of infants with presumed and confirmed infection were similar [25]. A peculiar aspect is the location of brain calcifications: the cortical–subcortical junction. This has been recognised as a typical feature of CZS [8,9,24]. Besides that, brain calcifications in this localisation have also been found in normocephalic infants with CZS [26]. The pattern and location of the brain calcifications may suggest a possible aetiology. Brain calcifications may be seen in other congenital infections, for example, cytomegalovirus and toxoplasmosis infection, but in these cases are predominantly periventricular [9,27]. This pattern of brain malformations in ZIKV is compatible with the fetal brain disruption sequence observed in congenital infections [28]. Experimental models showed that ZIKV targets human brain cells, reducing their viability and growth, suggesting that it abrogates neurogenesis during human brain development [29]. It has also been demonstrated that it infects the brain and the spinal cord, except the olfactory bulb. It has a high level of replication in the brain tissue, inducing cell death and significantly affecting the size and weight of the brain and, consequently, its development [30].

Neurological abnormalities were found in most of our cases: hypertonia/spasticity (97.0%), neurodevelopmental milestones delay (92.8%), and hyperreflexia (73.3%) suggest early signs of severe motor impairment. These findings are consistent with a clinical diagnosis of Cerebral Palsy (CP), which describes a group of permanent disorders of the development of movement and posture, causing activity limitation. The motor disorders of CP are often accompanied by disturbances of sensation, perception, cognition, communication, and behaviour [31]. Arthrogryposis was also found in our cases (10.2%). It possibly has a neurogenic origin due to the involvement of central and peripheral motor neurons, inducing fixed postures inside the uterus [11]. The majority of our cases presented epileptogenic activity (56.3%) or slow activity (8.3%) in the VEEG. These findings are likely related to the cortical malformations. One study estimated that approximately 54% of infants with clinical findings that are consistent with CZS have epilepsy, and that the prevalence of seizures increases with infant age and might not be associated with clinical findings [12]. Epilepsy was the main complication (48%) among CZS infants in the first 4 months of life, being the major cause of hospitalisation and emergency room visits [15].

We found impaired response in the ABR (17.3%) and VEP (14.1%), suggestive of sensorineural loss. The prevalence of sensorineural hearing loss was estimated as 5.8% among 69 infants with microcephaly and CZS [14]. These children need regular follow-ups, even the ones with normal initial screening tests, because hearing loss, like in other congenital viral infections, can be delayed and progressive. Severe visual impairment has been reported in all cases with CZS, as a consequence of retinal and/or optic nerve alterations [16]. In our cases, this may be a consequence of cortical and subcortical alterations.

The complaints reported by caregivers provide information on the challenges to be faced by caregivers of infants with CZS. These children require special health care in many aspects of development during their whole life. The prompt identification of potential disabilities enables early intervention and planning for resources to support these families, in health care and community settings, improving the quality of life for affected children and families.

It is important to highlight our limitations: Firstly, we acknowledge the absence of a laboratory confirmatory diagnosis of ZIKV infection. Unfortunately, the laboratory test to investigate ZIKV infection only became available in October 2016 in Brazil [32]. However, all included infants were born in an area affected by the ZIKV epidemic, and all mothers had clinical symptoms of ZIKV during pregnancy. All infants were assessed in regard to other potential causes of microcephaly. Therefore, the diagnosis of CZS was established on the basis of clinical and radiological criteria. Secondly, this study was of retrospective design and conducted in a single centre. Nonetheless, all children were evaluated by well-trained health professionals following standardised procedures, and data were registered in predefined forms. Thirdly, considering that the study was conducted in a referral centre, it is possible that children with more severe neurological symptoms were more likely to be referred to this centre for assessment, which may figure as a selection bias.

## 5. Conclusion

This group of infants who presented with microcephaly and whose mothers were exposed to ZIKV during pregnancy showed clinical and radiological criteria for CZS. A high frequency of brain abnormalities and signs of early neurological disorders were found, and epileptogenic activity and signs of sensorineural alterations were common. This suggests that microcephaly can be associated with a worst spectrum of neurological manifestations.

## Figures and Tables

**Table 1:** Characteristics of infants with microcephaly and probable CZS.

Findings	Mean	SD
At birth		
Gestational age, weeks	38.4	1.7
Weight, Kg	2.6	0.5
Length, cm	45.0	3.4
HC, cm	28.6	1.7
At admission		
Age, months	4.1	2.3
Weight, Kg	5.4	1.7
HC, cm	33.1	4.4
Age in which neurological exam was performed, months	4.6	2.4

**Table 2:** Demographic and perinatal characteristics of infants with microcephaly and probable CZS.

	Number of Evaluated Infants	Frequency (%)
Female	102	56 (54.9)
Severe microcephaly *	102	56 (54.9)
Premature ^†^ (34.2 ± 0.8 weeks)	102	09 (8.8)
Delivered by caesarean section	100	58 (58.0)
Apgar score 5 min between 7 and 10	84	82 (97.6)
Apgar score 5 min between 5 and 6	84	02 (2.4)
Neonatal complications ^‡^	100	38 (38.0)

* HC < −3 SD; ^†^ born alive before 37 weeks of pregnancy are completed; ^‡^ Jaundice (19.0%) and early seizures/respiratory distress (8.0%). CZS: Congenital Zika Syndrome.

**Table 3:** Evaluation findings in infants with probable CZS.

Evaluation Findings	Number of Evaluated Infants *	Frequency (%)
Neuroimaging *		
Cerebral atrophy	102	94 (92.1)
Ventriculomegaly	101	93 (92.1)
Malformation of cortical development	101	86 (85.1)
Location of calcifications		
• Cortical and subcortical	101	81(80.2)
• Basal ganglia	101	62 (61.4)
• Periventricular	101	30 (29.7)
• Brainstem	101	10 (9.9)
• Cerebellum	101	3 (2.9)
Corpus callosum abnormalities	102	76 (74.6)
Enlarged subarachnoid space	101	51 (50.5)
Cerebellum hypoplasia	101	24(23.7)
Brainstem hypoplasia	101	20 (19.8)
Enlarged cisterna magna	101	19 (18.8)
Delayed myelination	97	5 (5.1)
Intraparenchymal cysts	101	2 (1.9)
Videoeletroencephalogram		
Epileptogenic activity	96	54 (56.3)
Slow activity	96	8 (8.5)
Normal activity	96	34 (35.4)
Neurological findings		
Hypertonia/spasticity	101	98 (97.0)
Neurodevelopmental milestones delay	101	91 (92.8)
Hyperreflexia	101	74 (73.3)

* The number of evaluations was calculated according to the quality of the neuroimaging exams (CT scan, 72; MRI scan, 25; both, 5) for detection of the specific neuroimaging finding.
